# Pulsed Light Accelerated Crosslinking versus Continuous Light Accelerated Crosslinking: One-Year Results

**DOI:** 10.1155/2014/604731

**Published:** 2014-08-03

**Authors:** Cosimo Mazzotta, Claudio Traversi, Anna Lucia Paradiso, Maria Eugenia Latronico, Miguel Rechichi

**Affiliations:** ^1^Ophthalmic Operative Unit, Siena University Hospital, Siena, Italy; ^2^Santa Lucia Eye Center, Cosenza, Italy

## Abstract

*Purpose*. To compare functional results in two cohorts of patients undergoing epithelium-off pulsed (pl-ACXL) and continuous light accelerated corneal collagen crosslinking (cl-ACXL) with dextran-free riboflavin solution and high-fluence ultraviolet A irradiation. *Design*. It is a prospective, comparative, and interventional clinical study. *Methods*. 20 patients affected by progressive keratoconus were enrolled in the study. 10 eyes of 10 patients underwent an epithelium-off pl-ACXL by the KXL UV-A source (Avedro Inc., Waltham, MS, USA) with 8 minutes (1 sec. on/1 sec. off) of UV-A exposure at 30 mW/cm^2^ and energy dose of 7.2 J/cm^2^; 10 eyes of 10 patients underwent an epithelium-off cl-ACXL at 30 mW/cm^2^ for 4 minutes. Riboflavin 0.1% dextran-free solution was used for a 10-minutes corneal soaking. Patients underwent clinical examination of uncorrected distance visual acuity and corrected distance visual acuity (UDVA and CDVA), corneal topography and aberrometry (CSO EyeTop, Florence, Italy), corneal OCT optical pachymetry (Cirrus OCT, Zeiss Meditec, Jena, Germany), endothelial cells count (I-Conan Non Co Robot), and in vivo scanning laser confocal microscopy (Heidelberg, Germany) at 1, 3, 6, and 12 months of follow-up. *Results*. Functional results one year after cl-ACXL and pl-ACXL demonstrated keratoconus stability in both groups. Functional outcomes were found to be better in epithelium-off pulsed light accelerated treatment together with showing a deeper stromal penetration. No endothelial damage was recorded during the follow-up in both groups. *Conclusions*. The study confirmed that oxygen represents the main driver of collagen crosslinking reaction. Pulsed light treatment optimized intraoperative oxygen availability improving postoperative functional outcomes compared with continuous light treatment.

## 1. Introduction

Riboflavin UV-A induced corneal collagen crosslinking (CXL) represents a relatively new procedure available for the conservative treatment of progressive keratoconus [1, 2] and secondary corneal ectasia [3] due to its capacity in increasing biomechanical corneal resistance [4, 5] and intrinsic anticollagenase activity [6]. The physiochemical basis of crosslinking lies in the photodynamic types I-II reactions [7] induced by the interaction between 0.1% riboflavin molecules absorbed in corneal tissue and UV-A rays delivered at 3 mW/cm^2^ for 30 minutes (5.4 J/cm^2^ energy dose) releasing reactive oxygen species (ROS) that mediated crosslinks formation between and within collagen fibers [8, 9].

Conventional epithelium-off crosslinking (CXL) demonstrated its safety and long-term efficacy in stabilizing progressive keratoconus and secondary ectasias in different clinical trials [10–15]. On the other hand the procedure is time consuming lasting about 1 hour [16]. The Bunsen-Roscoe law of reciprocity [17–19] theoretically demonstrated that the photochemical process behind crosslinking depends on the absorbed UV-A energy and its biological effect is proportional to the total energy dose delivered in the tissue [17–19]. According to this concept it is theoretically possible to deliver the same energy dose ensuring a proportional biological effect by setting different UV-A powers and exposure times in order to accelerate and shorten the crosslinking procedure in accelerated crosslinking (A-CXL) modality [18–20]. According to photochemical crosslinking studies based on kinetics model [7] the UV-A illumination caused a rapid depletion of oxygen in a riboflavin soaked cornea and turning the UV light off led to replenishment of the oxygen to its original level within 3 to 4 minutes [7]. Krueger et al. [21] and Herekar [22] have also observed a rapid oxygen depletion during corneal crosslinking with riboflavin and concluded that the reactive oxygen species (ROS), specifically, singlet oxygen, are the predominant CXL reaction drivers. Under aerobic conditions, which are present during the first 10 to 15 seconds of UV-A exposure, sensitized photooxidation of the substrate (proteoglycan core proteins [23] and collagen in the corneal matrix) occurs mainly by its reaction with photochemically generated ROS, such as singlet molecular oxygen. This is consistent with a type II photochemical mechanism. After the first 10 to 15 seconds, oxygen becomes totally depleted and the reaction between the substrate and riboflavin becomes consistent with a predominantly type I photochemical mechanism [7]. Pulsing the UV light during crosslinking treatment theoretically restarts the photodynamic type II reaction achieving an additional oxygen concentration allowing more singlet oxygen release for crosslinking of collagen molecules. We report a comparative clinical study of continuous (cl-ACXL) and pulsed light (pl-ACXL) accelerated corneal collagen crosslinking in a series of 20 patients with progressive keratoconus investigating the functional outcomes at one-year follow-up and estimating the treatment penetration by means of in vivo confocal microscopy (IVCM).

## 2. Methods 

After specific informed consent subscription, 20 patients affected by progressive keratoconus were enrolled in the study. They were divided into 2 treatment groups: 10 eyes of 10 patients (pulsed light treatments), with age between 13 and 26 years (mean: 21.5 years), underwent an epithelium-off pulsed light accelerated corneal collagen crosslinking (pl-ACXL) by the KXL I UV-A source (Avedro Inc., Waltham, MS, USA) with 8 minutes (1 sec. on/1 sec. off) of UV-A exposure at 30 mW/cm^2^ with an energy dose of 7.2 J/cm^2^; 10 eyes of 10 patients (continuous light treatments), with age between 11 and 24 years (mean: 18,5 years), underwent an epithelium-off continuous light accelerated corneal collagen crosslinking (cl-ACXL) with the same instrument, UV-A power setting at 30 mW/cm^2^ for 4 minutes of continuous UV-A light exposure, and energy dose of 7.2 J/cm^2^. The riboflavin solution used in both treatment groups was composed of dextran-free riboflavin 0.1% with hydroxyl, propyl, methyl, and cellulose (VibeX Rapid, Avedro Inc., Waltham, MS, USA), with 10 minutes of corneal soaking. Treated eyes were dressed by a soft contact lens bandage for 3 days and medicated with ciprofloxacin eye drops, diclofenac, and sodium hyaluronate eye drops 4 times/day.

### 2.1. Inclusion Criteria

The parameters we considered to establish keratoconus progression and inclusion criteria for each group were worsening of UCVA/BSCVA > 0.50 Snellen lines, increase of SPH/CYL > 0.50 D, increase of topographic symmetry index SAI/SI >1 D, increase of mean* K* reading > 1 D, reduction of the thinnest point at corneal OCT pachymetry ≥10 *μ*m, and clear cornea at biomicroscopic examination. We considered “significant” for the inclusion in the study the variation of at least 3 of the parameters listed above (one clinical plus two instrumental).

### 2.2. Assessment Criteria

Pre- and postoperative examination included uncorrected distance visual acuity (UDVA), corrected distance visual acuity (CDVA), corneal topography simulated* K* average readings (*K* ave.), apical curvature (AK), and surface aberrometry (coma aberration) by CSO EyeTop Topographer (Costruzione Strumenti Oftalmici, Florence, Italy). In vivo scanning laser confocal microscopy was performed by the HRT II (Rostock Cornea Module, Heidelberg, Germany) and anterior segment OCT analysis by the Cirrus OCT instrument (Zeiss Meditec, Jena, Germany) in order to assess treatment penetration. Statistical analysis was performed using Wilcoxon test. All analyses were done using SPSS v16.0. A* P* value ≤ 0.05 was considered to be statistically significant.

## 3. Results

**Figure 1:**
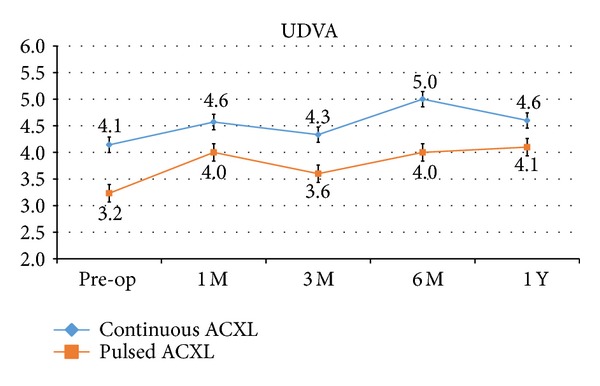
Uncorrected distance visual acuity (UDVA) after continuous light (blue line) and pulsed light (orange line) accelerated crosslinking gained +0.5 and +0.9 decimal equivalents, respectively, at one-year follow-up.

UDVA showed a statistically not significant improvement of +0.5, SD ± 1.2 (*P* = 0.65) and +0.9, SD ± 1.1 (*P* = 0.10) decimal equivalents in cl-ACXL and pl-ACXL, respectively, at one-year follow-up; see Figure 1.

**Figure 2:**
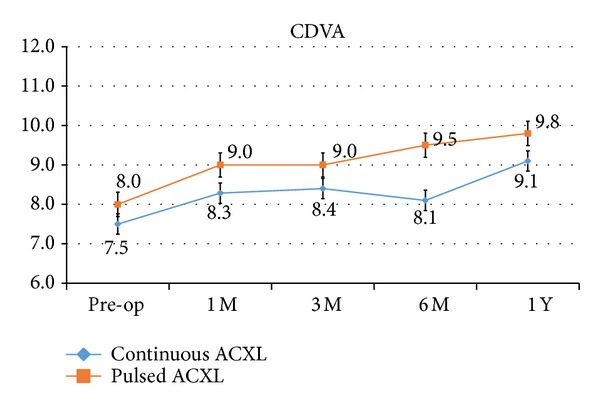
Corrected distance visual acuity (CDVA) after continuous light (blue line) and pulsed light (orange line) accelerated crosslinking gained +1.6 and 1.8 decimal equivalents on average, respectively, one year after treatment.

CDVA showed an improvement, even not statistically significant, in both groups by a mean value of +1.6 SD ± 1.0 (*P* 0.56) and 1.8 SD ± 1.3 (*P* = 0.55) decimal equivalents, respectively, one year after treatment; see Figure 2.

**Figure 3:**
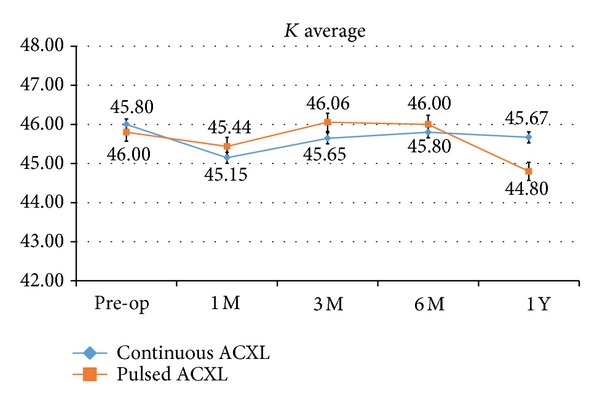
Topographic simulated* K* average (*K* ave.) value after continuous light (blue line) and pulsed light (orange line) accelerated crosslinking demonstrated a not statistically significant decrease by a mean value −0.13 Diopters; the reduction of* K* average was found to be statistically significant by a mean value of −1.2 Diopters after pulsed light accelerated CXL.

Topographic simulated* K* average value demonstrated a not statistically significant decrease one year after cl-ACXL by a mean value −0.13 Diopters, SD ± 0.13 (*P* = 0.088), while after cl-ACXL the reduction of* K* average was found to be statistically significant by a mean value of −1.2 Diopters, SD ± 0.4 (*P* = 0.049); see Figure 3.

**Figure 4:**
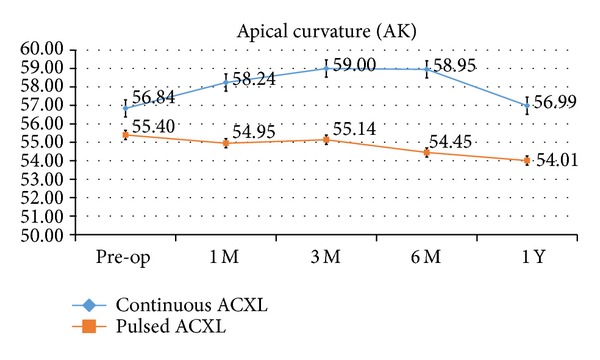
Topographic derived apical curvature value (AK) after continuous light (blue line) and pulsed light (orange line) accelerated crosslinking showed a statistically significant decrease in pulsed light accelerated CXL by a mean value −1.39 Diopters at one-year follow-up; no statistically significant differences were recorded in AK value after continuous light accelerated CXL.

Apical curvature value (AK) provided by the topographer showed a slight not statistically significant increase in cl-ACXL and a statistically significant decrease in pl-ACXL by a mean value of +0.15 Diopters, SD ± 0.8 (*P* = 0.077), and −1.39 Diopters, SD ± 0.38 (*P* = 0.05), respectively, at one-year follow-up; see Figure 4.

**Figure 5:**
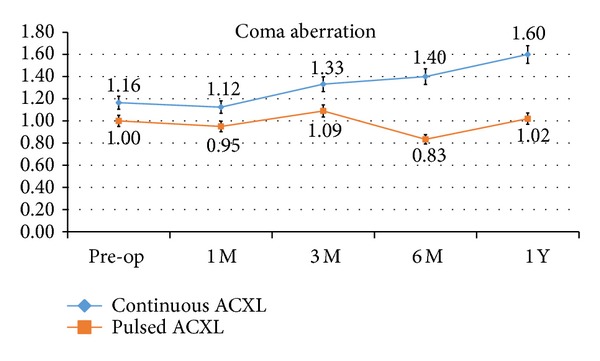
Coma values showed a statistically not significant difference one year after treatment. Continuous light accelerated crosslinking (blue line) was associated with a slight statistically not significant change of the coma by a mean value of +0.44 *μ*m, while pulsed light treatment (orange line) showed a stable value during the follow-up.

Coma aberration values showed a statistically not significant difference one year after treatment by a mean value of +0.44 *μ*m, SD ± 0,41 (*P* 0.58), in cl-ACXL and +0.02 *μ*m, SD ± 0,02 (0.068), in pl-ACXL; see Figure 5.

**Figure 6:**
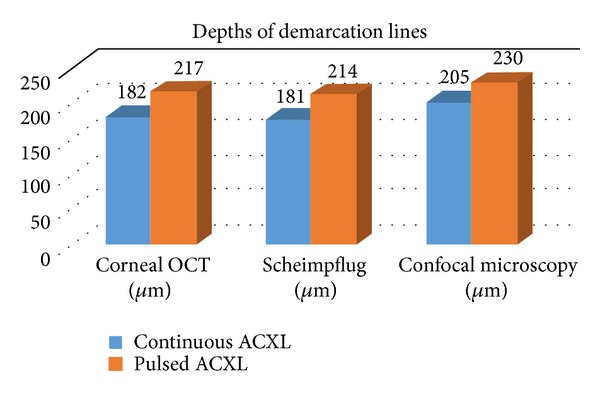
Depths of average demarcation lines recorded one month after continuous light (blue bar) and pulsed light (orange bar) accelerated crosslinking evaluated by corneal OCT (left), Scheimpflug camera (middle), and in vivo confocal microscopy (right) showing a deeper penetration of pulsed light treatment (orange bars) by a mean value of 215 *μ*m (±20 *μ*m) versus a lower penetration of continuous light treatment (blue bars) by a mean value of 160 *μ*m (±20 *μ*m).

**Figure 7:**
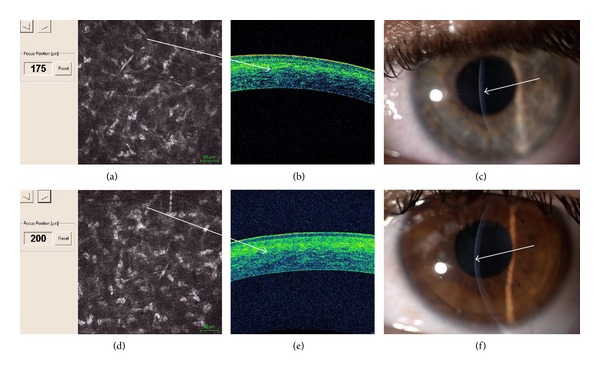
In vivo confocal microscopy (IVCM) after continuous light accelerated crosslinking showed keratocytes apoptosis until 175 *μ*m (up left (a)), an uneven demarcation line is well detectable with spectral domain corneal OCT showing hyperreflective corneal tissue (up, white arrow (b)); the demarcation line was also visible at slit lamp one month after treatment (up right, white arrow (c)). In vivo confocal microscopy (IVCM) after pulsed light accelerated crosslinking showed keratocytes apoptosis until 200 *μ*m (down left (a)); a deeper demarcation line is well detectable with spectral domain corneal OCT showing hyperreflective corneal tissue (down, white arrow (b)); the demarcation line was also visible at slit lamp one month after treatment (down right, white arrow (c)).

In vivo confocal microscopy (IVCM) after cl-ACXL showed an uneven demarcation line at mean depth of 160 *μ*m (range: 150–180 *μ*m) that was well visible one month after treatment. A deeper demarcation line was recorded after pl-ACXL at a mean depth of 200 *μ*m (range: 190–215 *μ*m) measured from the epithelial surface. A demarcation line was detectable at slit lamp examination; see Figures 6 and 7 and Table 1.

Preoperative mean endothelial cell density was 2450 cells/mm^2^ (range: 2082 to 3026 cells/mm^2^) in the cl-ACXL group and 2672 cells/mm^2^ (range: 2459–3016 cells/mm^2^) in the pl-ACXL group. Postoperative endothelial cells count at 12 months was 2355 cells/mm^2^ on average (range: 2172–2950 cells/mm^2^) in the cl-ACXL group and 2495 cells/mm^2^ (range: 2400–3125 cells/mm^2^) in pl-ACXL group.

No adverse events were recorded in both treatment groups during the follow-up.

## 4. Discussion

This comparative analysis, even if in a small case series, demonstrated the efficacy of continuous and pulsed light accelerated crosslinking in stabilizing keratoconus progression after one year of follow-up. Pulsed light treatment showed a slightly better functional outcome both in uncorrected and in corrected distance visual acuity even if there is no statistically significant difference between the two treatment modalities. UCVA was found to be slightly better in pl-ACXL patients and it may correlate with the statistically significant improvement of mean* K* values and reduction of apical curvature recorded in this cohort of patients. Conversely, there is no statistically significant difference in CDVA that improved in both groups at one-year follow-up. This slight difference could be attributed to the small number of the eyes included in the study. No adverse events were recorded in both treatment groups.

Pulsed light treatment showed a deeper apoptotic effect, meanly at 215 *μ*m of stromal depth (range: 190–235 *μ*m), while continuous light accelerated treatment revealed a penetration of 160 *μ*m on average (range: 150–180 *μ*m), both at confocal and at corneal OCT analysis as shown in Figures 6 and 7.

These findings were found to be slightly better than those recently reported in the literature [24] probably due to the higher energy dose used in our treatments (7.2 J/cm^2^ instead of 5.4 J/cm^2^) and pulsed light modality. Indeed, pulsing the UV-A light inducing an intraoperative oxygen reuptake while prolonging treatment time at 8 minutes may influence a deeper penetration of oxidative damage [25].

Accelerated corneal collagen crosslinking with pulsed and continuous UV-A light illumination reached the anterior part of the corneal stroma until 200 *μ*m of depth. This aspect assumes a physicochemical relevance because, as reported in the literature [26], the most important biomechanical effect related to crosslinking is concentrated in the anterior midstroma. Anyway the penetration of accelerated crosslinking remains under the value of conventional procedure (300 *μ*m) at 3 mW/cm^2^ for 30 minutes of UV-A exposure. Actually we do not know if this factor may negatively influence the biomechanical stability of keratoconus in a long-term follow-up. Conventional epithelium-off CXL procedure (riboflavin 0.1% plus dextran 20%, UV-A 3 mW/cm^2^ = 5.4 J/cm^2^ for 30 minutes) remains the gold standard in the conservative treatment of early-stage progressive keratoconus particularly in pediatric patients, even if, in our preliminary experience, the accelerated crosslinking with epithelium removal demonstrated its safety for endothelium both in pulsed and in continuous light treatment modality, shortening the CXL procedure time under 20 minutes, being well tolerated by patients. Pulsed light treatment seems slightly more capable to penetrate deeper in the corneal stroma compared to continuous light treatment giving better functional outcome even if in a limited case series. The functional improvement of accelerated CXL with pulsed energy could be traced back in an optimization of oxygen availability thanks to the on/off cycle of oxygen delivery. Anyway both treatments were found to have a similar efficacy in stabilizing keratoconus during the follow-up period. Pulsed and continuous light accelerated crosslinking represents safe evolving crosslinking procedures in order to achieve keratoconus stabilization in a short treatment time. The efficacy of these techniques still needs to be investigated in the mid-long-term follow-up and in a large cohort of patients.

## Figures and Tables

**Table 1:** Summary of the results.

	ΔUDVA (de)	ΔCDVA (de)	Δ*K* ave. (D)	ΔAK (D)	ΔComa (*µ*m)
Continuous light ACXL (cl-ACXL)	+0.5	+1.6	−0.13	+0.15	+0.44
Pulsed light ACXL (pl-ACXL)	+0.9	+1.8	−1.2	−1.39	+0.02

de: decimal equivalents; UDVA: uncorrected distance visual acuity; CDVA: corrected distance visual acuity; Δ*K* ave.: delta simulated *K* average reading; ΔAK (D): delta apical curvature; Δcoma: delta coma aberration.
